# Collector or Secretary?

**Published:** 1912-10-05

**Authors:** J. A. Rudd

**Affiliations:** Secretary. Coventry and Warwickshire Hospital, Coventry


### The Editor's Letter-Box.

### Collector or Secretary?

*To the Editor of* The Hospital.

Sir,—I wish to call your attention to a paragraph in last week's issue of your paper. On page 685 Mr. G. H. Hume is reported to have said : "In Newcastle we several times tried the expedient of a paid secretary, and have always given it up, because we found that it failed." I happened to be in the room at the time Mr. Hume was speaking, and distinctly remember that he said collector, and not secretary, as reported in your paper. I thought that perhaps you would be glad to have the error pointed out.—Yours faithfully,

J. A. Rudd, Secretary.

Coventry and Warwickshire Hospital,
Coventry, September 30.

[From inquiries among those present opinions conflict as to the actual word used. Perhaps Mr. Hume will decide the point, though the sense appears to demand the word "collector."—Ed. The Hospital.]

